# An Unsupervised Deep Learning Framework for Quantitative Breast Density Estimation from Mammograms

**DOI:** 10.3390/jimaging12070286

**Published:** 2026-06-29

**Authors:** Khaldoon Alhusari, Salam Dhou

**Affiliations:** Department of Computer Science and Engineering, American University of Sharjah, Sharjah P.O. Box 26666, United Arab Emirates; sdhou@aus.edu

**Keywords:** breast cancer, breast density estimation, mammography, segmentation, unsupervised learning

## Abstract

Breast cancer is the most commonly diagnosed cancer in women, with early detection playing a critical role in clinical outcomes. Mammography remains the standard screening modality, producing X-ray images used to assess mammographic density, a key indicator of the proportion of fibroglandular tissue within the breast. The Breast Imaging-Reporting and Data System (BI-RADS) classification system is widely used to report density across four qualitative categories. High density can obscure malignancies and is independently associated with elevated breast cancer risk. Manual interpretation of mammographic density is prone to subjectivity and inter-observer variability, and supervised learning-based estimation methods trained on subjective labels may reflect this inherent subjectivity. This work proposes an unsupervised framework for quantitative breast density estimation that requires no labeled data in its core pipeline. Expert labels are used exclusively to calibrate post hoc discretization thresholds for binary classification, enabling comparison with supervised methods in the literature. The main contributions include: (i) an adaptive Region of Interest (ROI) extraction algorithm, (ii) a Convolutional Neural Network (CNN) based unsupervised segmentation pipeline tuned for mammographic density separation, (iii) a novel confidence metric for identifying unreliable segmentation outputs, (iv) a label correction mechanism for low-confidence cases, and (v) a confidence-filtered majority voting scheme for per-patient classification. The framework is evaluated on two public datasets, namely DDSM and INbreast, with segmentation performance yielding Silhouette scores exceeding 0.92. Agreement with expert labels reaches 71.43% and 79.28% for DDSM and INbreast, respectively. Image-level clustering quality assessment confirms effective unsupervised labeling, with Silhouette scores averaging 0.57 for DDSM and 0.50 for INbreast. The proposed framework provides a practical and non-subjective model for quantitative breast density estimation, with potential utility as a decision-support tool for radiologists that can be considered in clinical practice after further investigation.

## 1. Introduction

**Figure 1:**
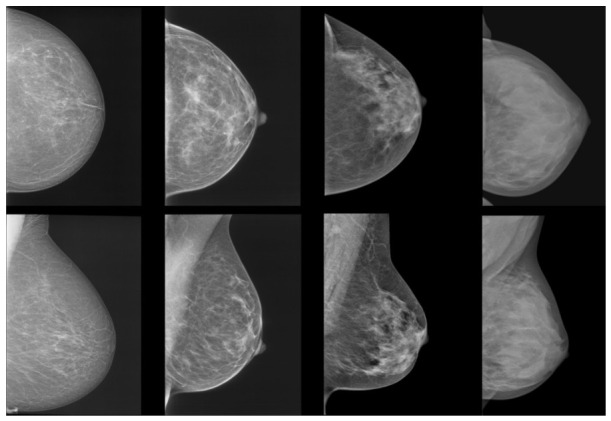
Sample CC (top row) and MLO (bottom row) mammograms with increasing breast density from left to right, ranging from almost entirely fatty (**left**) to almost entirely dense (**right**).

In 2022, breast cancer was the second most frequently diagnosed cancer type, with over 2.3 million cases reported across 185 countries [1]. Among females, it accounted for 23.8% of all cancer diagnoses and 15.4% of cancer-related fatalities. Treatment success depends heavily on early detection, with mammography the primary screening technique [2]. This method generates X-ray images of the breast, known as mammograms, that enable early identification of tumors, with a reported sensitivity of 86.9% [3]. Mammograms are also instrumental for radiologists in determining mammographic density, which refers to the proportion of radio-dense fibro-glandular (i.e., non-fatty) tissue in the breast [4]. High breast density is considered a significant risk factor for breast cancer, as studies indicate that women with denser breast tissue are substantially more likely to develop the disease [5]. On a mammogram, dense tissue appears white, while fatty tissue appears dark. Tumors and other abnormal growths also appear white, making them easier to detect in fatty breasts, where they contrast sharply. Conversely, in breasts with high density, the effectiveness of mammography decreases, as dense tissue may obscure tumors. Although mammography is a valuable tool, it is not without risks, as it involves exposure to radiation. While the probability of developing radiation-induced breast cancer is low, it is a factor to consider, particularly in women with larger, denser breasts [6,7,8]. Sample craniocaudal (CC) and mediolateral oblique (MLO) mammograms with varying breast density are provided in Figure 1 [9].

The effectiveness of mammography is largely dependent on the subjective judgment of the radiologist performing the interpretation. While radiologists typically concur on most cases, significant subjectivity emerges when evaluating highly dense breasts [10,11]. Additionally, it is not uncommon for cancers to be overlooked in initial mammogram readings and only detected during retrospective analyses [12]. These oversights may result from incorrect assessments of breast density, often caused by factors such as assuming asymmetrical distribution of fibro-glandular tissue. Misestimating breast density can also lead radiologists to make skewed clinical choices, including the recommendation of more invasive procedures. This occurs because mammogram sensitivity significantly declines in extremely dense breasts—with findings in [13] indicating it may drop to as low as 40%. Furthermore, cancers are often missed due to radiologists' cognitive biases, misreadings, and other contributing factors highlighted in [12]. Thus, while radiologist interpretation is generally reliable, it is not without limitations.

Accurate breast density estimation must also be devoid of subjectivity and uncertainty. However, most methods reported in the literature for estimating breast density rely on supervised learning trained on subjective expert annotations, introducing the very subjectivity they aim to eliminate. In our prior comprehensive review of machine learning approaches for breast density estimation [14], unsupervised segmentation was identified as a substantially underexplored direction, one with clear clinical motivation but limited systematic investigation. The present work directly addresses this gap.

In this work, we propose an unsupervised framework for quantitative breast density estimation that requires no labeled training data and no expert annotation at any stage in its core pipeline. The framework makes the following contributions: (i) an adaptive Region of Interest (ROI) extraction algorithm that determines region boundaries based on image content rather than fixed pixel dimensions; (ii) a CNN-based unsupervised segmentation pipeline, adapted and tuned from [15] specifically for mammographic density separation; (iii) a novel binary confidence metric, derived from intensity differences between segmented regions, to identify and handle cases where segmentation reliability is low; (iv) a predicted label correction mechanism for low-confidence cases, reassigning labels based on proximity to global dense and fatty intensity averages derived from high-confidence images; (v) a confidence-filtered majority voting scheme for aggregating image-level predictions into per-patient density classifications. A preprocessing pipeline for breast reorientation and artefact removal is also introduced to prepare mammograms for segmentation. The framework is evaluated on two public datasets, DDSM [16] and INbreast [17]. A preliminary version of this work has been reported [18].

## 2. Related Works

In our previous works [14,18,19], we have reviewed the literature surrounding breast density estimation in mammograms. The methods detailed can be classified into four categories: visual, software, machine learning, and segmentation-based methods.

Visual techniques are commonly applied to estimate breast density. These methods rely on parenchymal patterns and can be qualitative or semi-quantitative [20]. The BI-RADS system, developed by the American College of Radiology (ACR), is the most widely used qualitative framework [21]. It classifies breast density into four categories:

I. Fatty: almost no fibro-glandular tissue; composed mainly of fat
II. Scattered areas: small portions of fibro-glandular tissue
III. Heterogeneously dense: significant fibro-glandular tissue (>50%)
IV. Extremely dense: nearly all fibro-glandular tissue

Other visual schemes include Wolfe [22] and Tabár [23]. Both classify breasts into five categories based on parenchymal patterns. Some semi-quantitative visual approaches give numeric density approximations. Norman Boyd et al. defined six density ranges from visual estimates [5]. The Visual Analogue Scale is a 100 mm scale. It quantifies breast density from visual estimates with high accuracy [20,24].

Because visual assessment is subjective, software has been offered as a quantitative alternative. Some packages are semi-automatic and need a radiologist's input. Cumulus computes percent radiographic density from a radiologist’s annotation of dense-region edges [25]. Other packages are fully automatic. They require little or no radiologist effort. They are designed for easy integration. They follow either area-based or volume-based approaches. Area-based methods use 2-D area measures. Densitas is a notable area-based system. It uses two deep learning models: one estimates percent density, and the other provides a descriptive classification aligned with the four BI-RADS classes [20]. Volume-based methods estimate the physical volume of dense tissue. Quantra [26] and Volpara [27] are examples. They estimate breast volume, segment tissue by density, and compute percent dense tissue. They use different physics models. Volpara uses a relative physics model. Quantra uses an absolute physics model.

Machine learning has also been applied to density estimation in several works. These works propose mammogram preprocessing and feature extraction pipelines to improve performance. The methods utilized can be split into traditional machine learning and deep learning approaches. Most of these methods produce qualitative estimates that follow the BI-RADS classification scheme.

Preprocessing steps differ across studies, but several common trends can be observed. Region-of-interest (ROI) extraction is one of the most frequently used techniques, and studies that include ROI extraction generally report better performance [28]. The work in [29] suggests that the central region of the breast provides the most useful information for distinguishing between density categories. Several studies, including [28,30,31], describe methods for removing artefacts, embedded labels, and the pectoral muscle to improve image quality and analysis accuracy. In [32], the authors propose that all mammograms in a dataset should be oriented in the same direction to simplify later processing steps, and they introduce a method to achieve this. Additionally, a number of studies [33,34,35,36,37,38,39] mention that rescaling mammogram images before analysis can help speed up subsequent processing without significantly affecting estimation accuracy.

Traditional machine learning methods generally rely on hand-crafted feature extraction procedures, where statistical and/or textural features are extracted. For instance, the work in [28] extracted gray-level co-occurrence matrix (GLCM) and statistical features and fed them into support vector machine (SVM) and extreme learning machine (ELM) models, with a linear kernel SVM achieving the highest accuracy. The work in [30] extracted statistical features from ROI histograms and fed them into a feed-forward neural network. The best performance was achieved by a network with eight hidden layers. In [31], statistical features were fed into a directed acyclic graph SVM (DAG-SVM), achieving moderate accuracy. In [40], a large feature vector consisting of statistical and textural features was fed into a hierarchical classification model, which broke the 4-class problem down into three binary classification steps. The highest accuracy was achieved by a hybrid model that integrated a neuro-fuzzy classifier (NFC), an SVM, and a k-nearest neighbors (KNN) model, with each preceded by Principal Component Analysis (PCA). The work in [41] utilized GLCM and histogram features with KNN and Naïve Bayesian models, with 1-NN performing best. Finally, the work in [42] fed histogram features into an SVM, which split input images into high- and low-density regions, and employed arithmetic division to produce numeric density estimates. The estimates were then used to classify mammograms with notable accuracy.

In contrast, deep learning methods can learn complex features from input mammograms independently. The work in [33] introduces a conditional generative adversarial network (cGAN) followed by a CNN to binarize mammograms and then classify them into one of four density categories, achieving significant performance. In [34], a deep CNN comprising 13 convolutional layers was implemented and exhibited high accuracy. A lightweight CNN with three convolutional layers was introduced in [35] and demonstrated notable classification accuracy. A transfer learning approach was employed in [36], wherein the Inception_ResNet_V2 model was fine-tuned for breast density assessment, resulting in impressive accuracy. The work in [37] leveraged a deep learning architecture combining ResNets with adaptive spatial and channel attention modules, dubbed BASCnet, and reported considerable density assessment performance. A very deep residual CNN with 41 convolutional layers was used in [38], and notable accuracy was observed. The authors of [39] added dilated convolution and channel-wise attention layers to the ResNet architecture originally proposed in [43] and applied it to the problem of breast density assessment. In [44], three CNN models were enhanced with squeeze-and-excitation (SE) attention blocks, with the augmented Inception_V4 model proving to be the most effective for density classification. Finally, in [45], transfer learning was applied with a deep CNN that was previously trained in [46] for cancer screening, and high accuracy was attained.

The machine learning methods described earlier are supervised and depend on subjective expert labels for training. By contrast, mammogram segmentation offers a mainly unsupervised alternative in which the breast region is segmented to produce a quantitative measure of overall density that can be used for cancer risk assessment in mammograms [47]. Automatic density-segmentation methods are commonly divided into two classes: area density projection–based methods and volume density projection–based methods. Area projection approaches operate on the 2-D image and include techniques such as thresholding, clustering, statistical modeling, and collective multiple-measurement schemes to separate dense from non-dense tissue. Volume projection approaches attempt to estimate the depth or thickness of tissue represented in the image so that a true volume can be computed; these methods rely on prior calibration, in-image reference calibration, or turnkey software solutions such as Quanta and Volpara.

Segmentation methods have been applied directly to breast density assessment to produce continuous percent density estimates by following the segmentation process with arithmetic division. In [48], multiple unsupervised segmentation steps, including Gaussian mixture modeling and K-means, were incorporated to segment and estimate breast density. The method described in [4] combines unsupervised feature learning with supervised classification, utilizing a convolutional sparse autoencoder (CSAE) to learn features from unlabeled mammographic images and a softmax regression classifier to estimate the probability of each pixel belonging to the dense class. The authors of [49] presented a supervised multitask deep learning model with an encoder–decoder architecture. The model was trained using expert-annotated segmentation masks and produced density estimates that correlated very well with radiologist assessments.

Despite the breadth of methods surveyed, a clear gap remains. The overwhelming majority of machine learning approaches for breast density estimation are supervised, relying on expert-annotated labels that are inherently subjective and costly to obtain. Among unsupervised alternatives, existing segmentation-based methods either employ primitive techniques such as K-means and Gaussian mixture modeling [48] or combine unsupervised feature learning with a supervised classification stage [4], falling short of a fully annotation-free pipeline. Deep learning-based unsupervised segmentation, despite its potential, has not been systematically applied to breast density estimation. Furthermore, no prior work has addressed the reliability of unsupervised segmentation outputs through a confidence-aware correction and aggregation mechanism. The present work addresses these gaps directly, proposing an unsupervised deep learning framework that requires no labeled data in its core pipeline, and introducing novel mechanisms for segmentation confidence assessment, label correction, and per-patient density aggregation. A quantitative comparison of the proposed framework with contemporary methods is provided in Table 8 in Section 5.2.

## 3. Methodology

### 3.1. Datasets

Two datasets are used to evaluate the framework proposed in this study: DDSM [16,50] and INbreast [17]. This section presents an analysis of the two datasets, summarized in Table 1.

The Curated Breast Imaging Subset of DDSM (CBIS-DDSM) comprises 1894 publicly accessible images, with 902 CC images and 992 MLO images. After the selection and preprocessing phases, 1050 images remain, corresponding to 805 patients. Among these patients, 593 have only one mammogram, 212 have at least two, and 12 have all four (left CC, right CC, left MLO, and right MLO) available. Regarding labels, 549 images are classified as fatty (BI-RADS I or II), and 501 as dense (BI-RADS III or IV).

The INbreast dataset comprises 410 images from 115 patients, with 203 CC images and 206 MLO images. Following selection and preprocessing, 309 images remain, attributed to 111 patients. Among these, 104 patients have at least two images, while 41 have exactly four, and 7 have only one. Regarding labels, 210 images are classified as fatty, and 99 as dense.

For both datasets, images are initially in DICOM format. DICOM files were read using pydicom (v2.3.0), with raw pixel arrays extracted without intensity windowing or normalization, and saved as JPEG images using matplotlib (v3.8.0). No explicit bit-depth conversion, dynamic range adjustment, or compression settings were applied beyond the default behavior of the respective libraries.

### 3.2. Mammogram Selection and Preprocessing

Given that tumors often resemble dense tissue in mammograms, only negative mammograms (non-malignant cases) are chosen for this work. Both MLO and CC mammograms are utilized, with predictions made for individual images and subsequently aggregated for per-patient assessment. Prior to preprocessing, images were downsampled by a factor of 5 in both spatial dimensions to reduce computational load.

**Figure 2:**
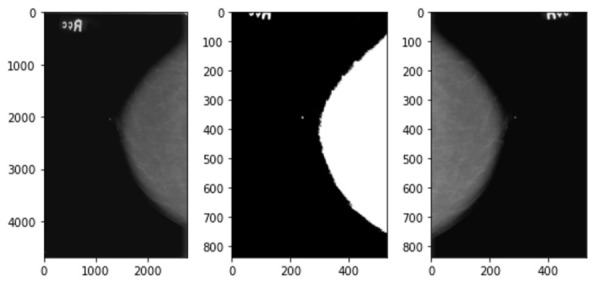
Sample of breast reorientation to face right, applied to a CC mammogram from DDSM.

It is also important that all breasts in a set of mammograms face the same direction. To standardize the orientation of breasts, an approach inspired by the method detailed in [31] is applied, wherein the mammogram is first binarized and then split horizontally. The pixel values of the split sides of the binarized image, amounting to 1′s and 0′s, are added up for each side. The side with the higher sum is the one that contains the breast. If a reorientation is required, the original (non-binarized) mammogram is flipped along the *y*-axis. As a standard, the images are all oriented to be east/right-facing. A sample is provided in Figure 2.

**Figure 3:**
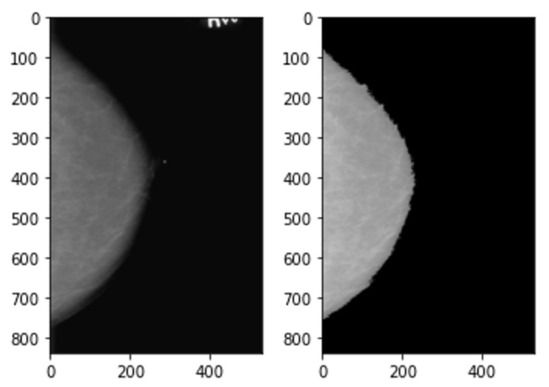
Sample of artefact removal, applied to a CC mammogram from DDSM.

**Figure 4:**
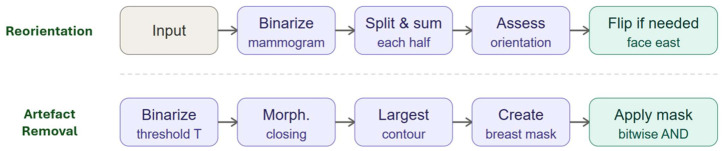
Flowchart of the reorientation and artefact removal preprocessing pipelines.

Mammograms often contain artefacts that can hinder segmentation. In this work, to eliminate these artefacts, in each mammogram, the breast is isolated, and everything else is considered background and given an intensity of zero (black). The images are first binarized again, this time with a higher threshold intensity of 30 for DDSM and 70 for INbreast (out of a maximum pixel value of 1000). Morphological closing is applied to the binary images using an elliptical structuring element of size 5 × 5 to make sure each apparent island, breast tissue included, is fully connected. Then, the largest contour in the binary image, presumably the breast, is used as a mask and applied to the original mammogram through a bitwise AND operation. This process leaves a mammogram with all artefacts removed without making any changes to the original shape of the breast or the size of the image. A sample is provided in Figure 3. A flowchart of the reorientation and artefact removal pipelines is shown in Figure 4.

**Figure 5:**
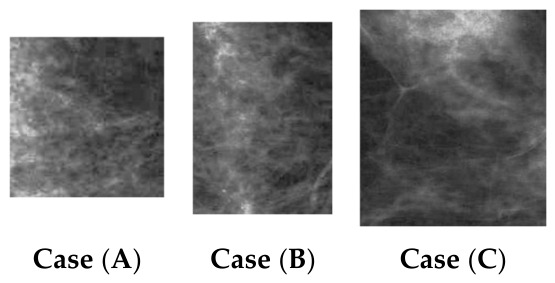
Samples of ROIs extracted from CC mammograms from DDSM. (**A**) Sample 1; (**B**) Sample 2; (**C**) Sample 3.

According to [29], the central region of the breast is the most indicative of differences between distinct breast density categories. In [40], ROIs with a fixed size of 128 × 128 pixels performed best. However, 128 × 128 pixels is too rigid. This work introduces an ROI extraction algorithm with adaptive sizes. The algorithm, showcased in Algorithm 1, works by finding four corner coordinates and extracting the rectangle between them. The horizontal boundaries (p1, p3 = 0.1; p2, p4 = 0.7 of the breast width) and vertical boundaries (1.2 and 0.8 times the first nonzero y-coordinate at the right x-coordinate) were chosen to consistently capture central breast tissue across both datasets. Samples of extracted ROIs are provided in Figure 5.
**Algorithm 1:** ROI ExtractionInput:  Mammograms—a set of preprocessed mammogram images Output: Rectangular ROIs with adaptive sizes
1: for each mammogram in mammograms do 2:     binarizedMammogram ← Binarize(mammogram) 3:     xBoundary ← max(x: binarizedMammogram[x] > 0) 4:     mammogram ← Crop(mammogram, x = [0, xBoundary]) 5:     x_topLeft     ← p1 × xBoundary 6:     x_topRight    ← p2 × xBoundary 7:     x_bottomLeft  ← p3 × xBoundary 8:     x_bottomRight ← p4 × xBoundary 9:     y_top    ← min(y: binarizedMammogram[y, x_topRight] > 0) 10:    y_bottom ← max(y: binarizedMammogram[y, x_topRight] > 0) 11:    corners ← {(x_topLeft, y_top), (x_topRight, y_top),                              (x_bottomLeft, y_bottom), (x_bottomRight, y_bottom)} 12:    ROI ← Crop(mammogram, corners) 13:    Save(ROI) 14: end for

**Figure 6:**
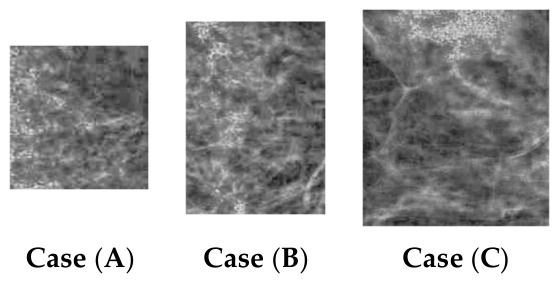
The ROIs from Figure 5 with CLAHE applied. (**A**) Sample 1; (**B**) Sample 2; (**C**) Sample 3.

Mammograms often suffer from blurriness, which can affect density perception. In this work, Contrast Limited Adaptive Histogram Equalization (CLAHE) is applied to the ROIs directly before image segmentation, in order to make different density regions in the ROIs more distinctive. The same ROIs from Figure 5 are shown in Figure 6 following the application of CLAHE.

### 3.3. Unsupervised Mammographic Density Segmentation

Mammogram segmentation is an essential step for breast density estimation. In this work, unsupervised segmentation is used as an alternative to traditional machine- or deep-learning-based classification. Unlike supervised methods, unsupervised segmentation does not depend on labeled data, instead leveraging the data’s inherent structure to detect patterns and group similar elements [15]. In contrast with the preprocessing steps in Section 3.2, which isolate the breast region from the background, this stage segments the breast tissue itself into dense and fatty regions to facilitate density estimation.

A CNN-based general-purpose, unsupervised image segmentation method is used [15] to segment ROIs of mammograms into two clusters to facilitate percent density estimation and subsequent binary classification. The network includes a modifiable number of convolutional layers (minimum of two) with batch normalization and ReLU activations. It outputs segmentation masks that separate images into regions of similar pixels by minimizing a combination of similarity loss and spatial continuity loss. The algorithm iterates until a minimum label count, the number of clusters in the segmented image, or a maximum number of iterations is reached. Importantly, the model is initialized and trained independently for each input image until convergence, rather than being trained once across the dataset. As such, no conventional train/validation/test split is applied to the segmentation network itself. The loss function is defined as follows:

(1) $$L=L_{sim}\left(\left\{{r^{\prime }}_{n},C_{n}\right\}\right)+{\mu L}_{con}\left(\left\{{r^{\prime }}_{n}\right\}\right)$$

Here, $L_{sim}$ is the similarity loss, $L_{con}$ is the continuity loss, ${r^{\prime }}_{n}$ is the normalized response map, $C_{n}$ the cluster labels, and μ a weight balancing the two terms. Cross-entropy is used for $L_{sim}$, as testing confirmed it to be the best-performing similarity loss for this task:

(2) $$LL_{sim}\left(\left\{{r^{\prime }}_{n},C_{n}\right\}\right)=\sum_{n=1}^{N}\sum_{i=1}^{q}-\delta \left(i-C_{n}\right)\ln{r^{\prime }}_{n,i},$$

where *N* is the number of pixels in the response map, *q* is the number of cluster labels for each pixel, and *δ* is a function of t defined as:

(3) $$\delta \left(t\right)=\left\{\begin{array}{c} 1,\;\;\;\;if\;t=0\\ 0,\;\;otherwise. \end{array}\right.$$

For $L_{con}$, the original algorithm used L1 loss (MAE). However, tests showed that mean squared error (MSE) performed better on INbreast. Therefore, both were used in this work. Specifically, MAE is used as the continuity loss for DDSM, and MSE for INbreast:

(4) $$L_{con(MAE)}\left(\left\{{r^{\prime }}_{n}\right\}\right)=\sum_{\varepsilon =1}^{W-1}\sum_{\eta =1}^{H-1}\left\|{r^{\prime }}_{\varepsilon +1,\eta }-{r^{\prime }}_{\varepsilon ,\eta }\right\|+\left\|{r^{\prime }}_{\varepsilon ,\eta +1}-{r^{\prime }}_{\varepsilon ,\eta }\right\|$$

(5) $$L_{con(MSE)}\left(\left\{{r^{\prime }}_{n}\right\}\right)=\sum_{\varepsilon =1}^{W-1}\sum_{\eta =1}^{H-1}{\left({r^{\prime }}_{\varepsilon +1,\eta }-{r^{\prime }}_{\varepsilon ,\eta }\right)}^{2}+{\left({r^{\prime }}_{\varepsilon ,\eta +1}-{r^{\prime }}_{\varepsilon ,\eta }\right)}^{2}$$

For both Equations (4) and (5), W and H represent the image width and height, and ${r^{\prime }}_{\varepsilon ,\eta }$ is the pixel value at $\left(\varepsilon ,\eta \right)$ in the response map ${r^{\prime }}_{n}$.

**Figure 7:**
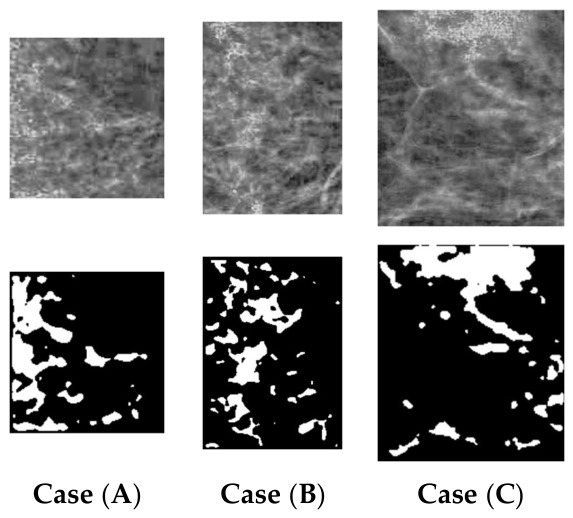
The CLAHE-applied ROIs from Figure 6 alongside their segmentation results. In the segmented images, the white areas are the dense regions, and the black areas are the fatty regions of the breast. (**A**) Sample 1; (**B**) Sample 2; (**C**) Sample 3.

The algorithm uses backpropagation. An input image is passed through the CNN to produce a response map with pixel-wise label probabilities. The most probable label (argmax) is assigned to each pixel to form clusters, and loss is computed from the final labels. During training, the model parameters are updated via Stochastic Gradient Descent (SGD) using gradients computed through backpropagation. The process continues until either convergence (minimum of 2 labels) or the maximum iteration count (default 1000) is reached. A sample of the segmentation results is provided in Figure 7.

### 3.4. Breast Density Estimation

For the task of breast density estimation, continuous percentage density estimates can be computed given the segmented image masks using arithmetic division. Specifically, the segmented mammogram is treated as a binary image comprising two regions. The region with the higher mean intensity in the original mammogram is taken as the dense region, and the other as fatty. Accordingly, a dense pixel is defined as any pixel belonging to the higher-intensity segmented region. The percentage density is then calculated by dividing the number of dense pixels by the total pixel count across both regions. This is expressed mathematically as:

(6) $$\%\;Density=\;\frac{\#\;of\;Dense\;Pixels\;\times 100}{\#\;of\;Dense\;Pixels\;+\;\#\;of\;Fatty\;Pixels}$$

**Figure 8:**
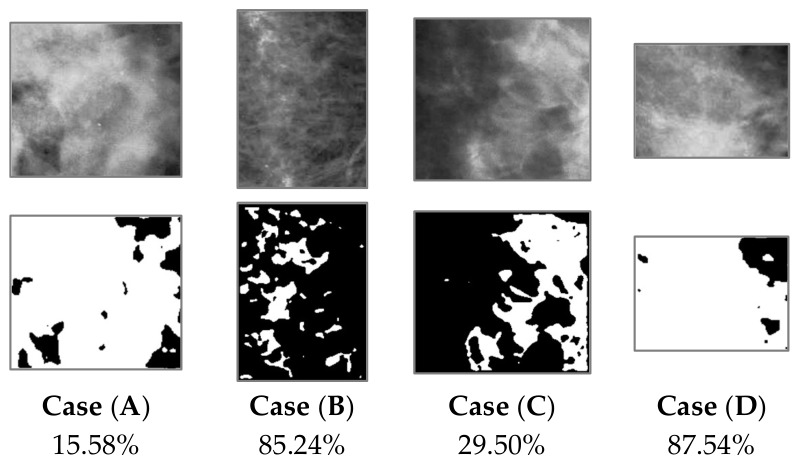
Samples of segmented images with their continuous density estimates. (**A**) Case A, density estimate: 15.58%; (**B**) Case B, density estimate: 85.24%; (**C**) Case C, density estimate: 29.50%; (**D**) Case D, density estimate: 87.54%.

These resulting estimates are either used directly as quantitative measures or transformed into qualitative categories. The aim of this study is to generate density estimates in an unsupervised fashion, reflecting a clinical scenario where prior density labels are not available. Accordingly, assigning qualitative density labels is not a core feature of the proposed framework. Nonetheless, for the framework to be clinically viable, it must demonstrate substantial agreement with expert annotations in the majority of cases. To evaluate this, the continuous percentage estimates are also discretized into binary qualitative labels, while the continuous values are retained as quantitative outputs, and the four-category expert labels are binarized as well, grouping BI-RADS I and II into a fatty class and BI-RADS III and IV into a dense class. Algorithm 2 describes this process. It is important to note that α governs the discretization of already-computed density estimates into binary qualitative labels. It has no influence on the continuous percentage density estimates themselves. A sample of the resulting density estimates is provided in Figure 8.
**Algorithm 2:** Density Estimation and DiscretizationInput:  segmentedMasks—a set of binary segmented image masks,               mammograms—the corresponding original mammograms Output: Continuous density estimates, binary qualitative labels,               and region averages {avgDense, avgFatty} per image
1: densities ← [] 2: regionAverages ← [] 3: for each (mask, mammogram) in (segmentedMasks, mammograms) do 4:     avgIntensity1 ← MeanIntensity(mammogram, mask, region = 1) 5:     avgIntensity2 ← MeanIntensity(mammogram, mask, region = 2) 6:     if avgIntensity1 ≥ avgIntensity2 then 7:         densePx ← CountPixels(mask, region = 1) 8:         fattyPx ← CountPixels(mask, region = 2) 9:         avgDense ← avgIntensity1 10:        avgFatty ← avgIntensity2 11:    else 12:        densePx   ← CountPixels(mask, region = 2) 13:        fattyPx   ← CountPixels(mask, region = 1) 14:        avgDense ← avgIntensity2 15:        avgFatty ← avgIntensity1 16:    end if 17:    density ← (densePx/(densePx + fattyPx)) × 100 18:    Append(densities, density) 19:    Append(regionAverages, {avgDense, avgFatty}) 20: end for 21: μ_d ← Mean(densities) 22: σ_d ← StdDev(densities) 23: threshold_d ← μ_d    − α × σ_d {α empirically optimized per dataset and view} 24: for each density in densities do 25:    if density ≤ threshold_d then 26:        label ← FATTY 27:    else 28:        label ← DENSE 29:    end if 30:    Save(density, label) 31: end for 32: Save(regionAverages)

### 3.5. Segmentation Confidence Assessment

Postprocessing the data is necessary to account for any faults that are inherent to using an unsupervised segmentation algorithm as the backbone of the framework. In some instances, breasts can appear to be almost entirely homogeneous in terms of density. However, regardless of the homogeneity of an image, the algorithm might still identify two clusters within it. This work introduces a qualitative confidence metric to identify and deal with such cases. It is noteworthy that the vast majority of images should have a “Confident” assignment, as the issue of homogeneity is relatively rare. Algorithm 3 describes this process. β governs confidence assignment as a post hoc step and does not influence the underlying segmentation or density estimation pipeline.
**Algorithm 3:** Confidence Metric AssignmentInput:  regionAverages—{avgDense, avgFatty} per image from Algorithm 2 Output: Confidence ∈ {Confident, Not Confident} per image
1: differences ← [] 2: for each image in regionAverages do 3:     diff ← |regionAverages[image].avgDense − regionAverages[image].avgFatty| 4:     Append(differences, diff) 5: end for 6: μ_c ← Mean(differences) 7: σ_c ← StdDev(differences) 8: threshold_c ← μ_c − β × σ_c    {β empirically optimized per dataset and view} 9: for each diff in differences do 10:    if diff ≤ threshold_c then 11:        confidence ← Not Confident 12:    else 13:        confidence ← Confident 14:    end if 15:    Save(confidence) 16: end for

### 3.6. Predicted Label Correction

Predicted label correction is applied exclusively to images assigned a “Not Confident” label. For such images, the segmentation output is considered unreliable, and the overall mean intensity of the image is used instead to determine the corrected label. Specifically, the mean intensity is compared against two global reference values: the global average dense intensity and the global average fatty intensity, both derived from confident images as identified in Section 3.5. The image is reassigned to whichever class its mean intensity more closely resembles. Algorithm 4 describes this process.
**Algorithm 4:** Predicted Label CorrectionInput:  images—images assigned Not Confident             globalAvgDense—mean Dense AVG Intensity across                                    Confident images, as identified by Algorithm 3                                    using regionAverages from Algorithm 2            globalAvgFatty—mean Fatty AVG Intensity across                                     Confident images, as identified by Algorithm 3                                    using regionAverages from Algorithm 2            predictedLabels—original label per image from Algorithm 2 Output: Corrected qualitative labels
1: for each image in images do 2:     avgIntensity ← MeanIntensity(image) 3:     if |avgIntensity − globalAvgFatty| ≤                |avgIntensity − globalAvgDense| then 4:          correctedLabel ← FATTY 5:     else 6:          correctedLabel ← DENSE 7:     end if 8:     if correctedLabel ≠ predictedLabels[image] then 9:          Save(correctedLabel) 10:    else 11:          Save(predictedLabels[image]) 12:    end if 13: end for

### 3.7. Per-Patient Density Assessment

Following confidence assessment and label correction, a per-patient density classification is determined using a majority vote over individual image predictions. Only images assigned a “Confident” label contribute to the vote. If a patient has no confident images, the confidence metric is disregarded, and all available predictions are used instead. In the event of a tie, the Dense label is assigned, reflecting the class imbalance towards Fatty observed in both datasets. Since the majority of expert assessments are Fatty, a tied vote is more likely to indicate a genuinely ambiguous Dense case than a misclassified Fatty one. In addition, this choice also reflects the clinical asymmetry of density misclassification. Underdetecting high density carries greater risk than over-reporting it, as dense tissue can mask malignancies. Algorithm 5 describes this process.
**Algorithm 5:** Per-Patient Density AssessmentInput:  patientImages—images grouped by patient, with              {Amended Prediction, Confidence} per image              from Algorithms 3 and 4 Output: Per-patient density label ∈ {Fatty, Dense}
1: for each patient in patientImages do 2:     count_f ← 0 3:     count_d ← 0 4:     allPredictions ← [] 5:     for each image in patient do 6:          Append(allPredictions, image.AmendedPrediction) 7:          if image.Confidence = Confident then 8:               if image.AmendedPrediction = FATTY then 9:                    count_f ← count_f + 1 10:               else 11:                    count_d ← count_d + 1 12:               end if 13:          end if 14:     end for 15:     if count_f = 0 and count_d = 0 then 16:          assessment ← FATTY if Mean(allPredictions) ≤ 1.5 else DENSE 17:     else if count_f > count_d then 18:          assessment ← FATTY 19:     else 20:          assessment ← DENSE 21:     end if 22:     Save(patient, assessment)

### 3.8. Model Evaluation

To qualify the effectiveness of the proposed framework, it is evaluated in four phases: a histogram-based labeling quality analysis, a segmentation evaluation at the pixel level, a performance assessment according to traditional classification metrics, and an assessment of unsupervised clustering quality at the image level. The metrics used aim to provide a comprehensive understanding of the framework’s performance.

For the first phase, an evaluation of the labeling quality of the framework is presented. For the analysis, histograms are used to compare the true and predicted labels based on four extracted features: luminance, entropy, skewness, and kurtosis. The second phase of evaluation assesses the quality of image segmentation at the pixel level. This employs the silhouette coefficient (SC), the Within-Cluster Sum of Squares (WCSS), the Davies–Bouldin (DB) score, and the Calinski–Harabasz (CH) index, defined as follows:

(7) $$SC=\frac{B-A}{max(A,B)\;}\;,$$

where A is the mean intra-cluster distance and B is the mean nearest-cluster distance. Higher SC values indicate better-defined clusters.

(8) $$WCSS=\sum_{i=1}^{k}\sum_{x\in C_{i}}{\left\|{x-u}_{i}\right\|}^{2},$$

where k is the number of clusters, $C_{i}$ is the i-th cluster, and $u_{i}\;$its centroid. Lower values indicate improved segmentation.

(9) $$DB=\frac{1}{k}\sum_{i=1}^{k}{max}_{i\neq j}\left(\frac{S_{i}+S_{j}}{D_{ij}}\right),$$

where $S_{i}$ and $S_{j}$ are the mean distances between points in clusters i and j and their respective centroids, and $D_{ij}$ is the distance between centroids i and j. Lower values indicate improved segmentation quality.

(10) $$CH=\frac{B}{W}\times \frac{N-k}{k-1},$$

where B and W are the between- and within-cluster variances, respectively, N is the total number of data points, and k is the number of clusters. Higher values suggest better segmentation.

The third stage of evaluation involves the calculation of accuracy, precision, recall, F1-score, and receiver operating characteristic (ROC) curve area under the curve (AUC), using expert labels as ground truth. In the fourth and final step, unsupervised clustering quality at the image level is assessed using SC, DB, and CH. These metrics are calculated at the per-image level, considering “Dense” and “Fatty” images as separate clusters. To facilitate clustering quality assessment, nine features are extracted from input ROIs: mean luminance, standard deviation, entropy, intensity ranges, 25th, 50th, and 75th percentiles, skewness, and kurtosis.

## 4. Results

In this section, results are presented for experiments performed on each of the two datasets, DDSM and INbreast. A summary of the results is provided in Table 2, and the optimized values of α and β are reported in Table 3. The results of the segmentation evaluation at the pixel level are shown in Table 4. Table 5 presents the results of the classification of mammograms into Fatty and Dense classes. The percentage agreement with the ground truth for each BI-RADS class is shown in Table 6. Table 7 showcases the results of the clustering evaluation at the image level. Grid search was used to identify the optimal combination of hyperparameters: number of convolutional layers (nConv), similarity loss step size (stepsize_sim), and continuity loss step size (stepsize_con). Due to the computational cost of running the segmentation model across full datasets, hyperparameter tuning was conducted on a randomly selected subset of 50 images per dataset and view. While this subset is drawn from the same data used for final evaluation, the influence of label-informed tuning is limited to the post hoc discretization of continuous density estimates into binary labels. It does not affect the segmentation or density estimation pipeline itself. Accordingly, the reported classification performance reflects within-dataset calibrated results. The starting number of channels (nChannel) was initially tested but fixed at 4 for DDSM-CC and 5 for all other subsets to speed up segmentation without sacrificing reliability. The learning rate (lr) was set to 0.10 following initial testing. A total of 48 hyperparameter combinations were evaluated across the CC and MLO subsets of both DDSM and INbreast, amounting to 192 runs, not including reruns or additional tests. Random seeds were fixed at 0 throughout all experiments to ensure reproducibility. The optimized values of α and β for each dataset and view are reported in Table 3.

### 4.1. Label Quality Analysis

**Figure A1:**
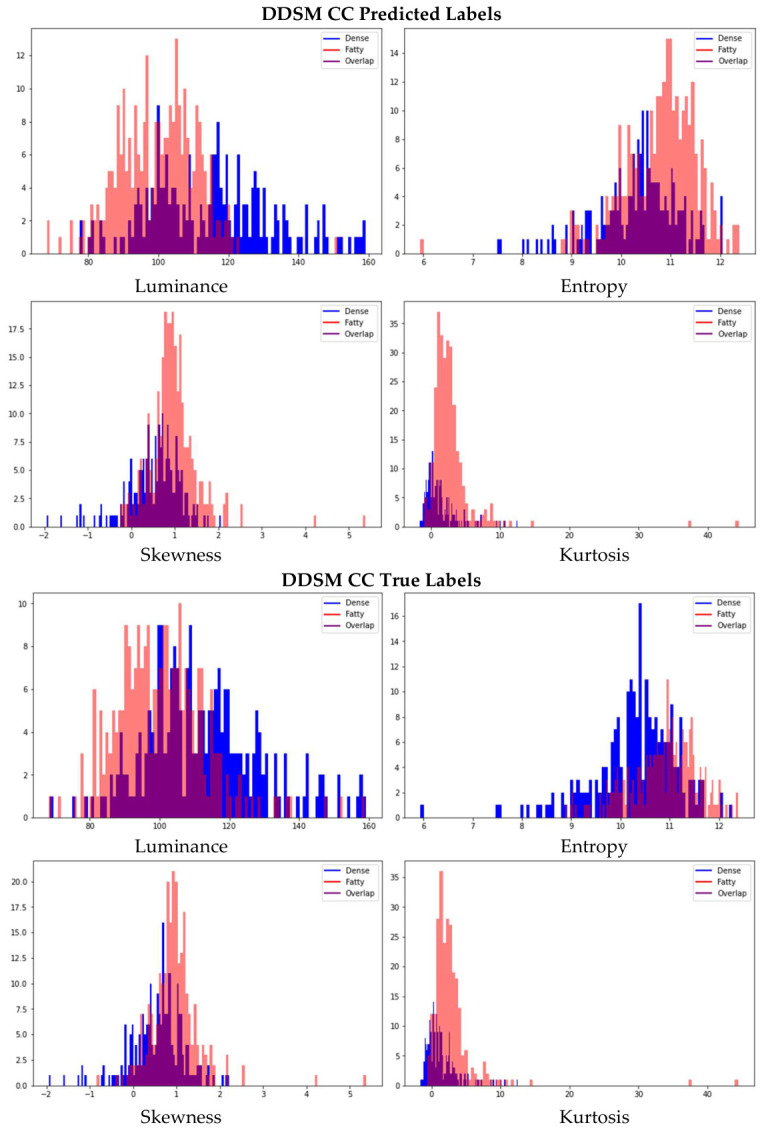
Label Quality Analysis Histograms for CC Images of the DDSM Dataset.

**Figure A2:**
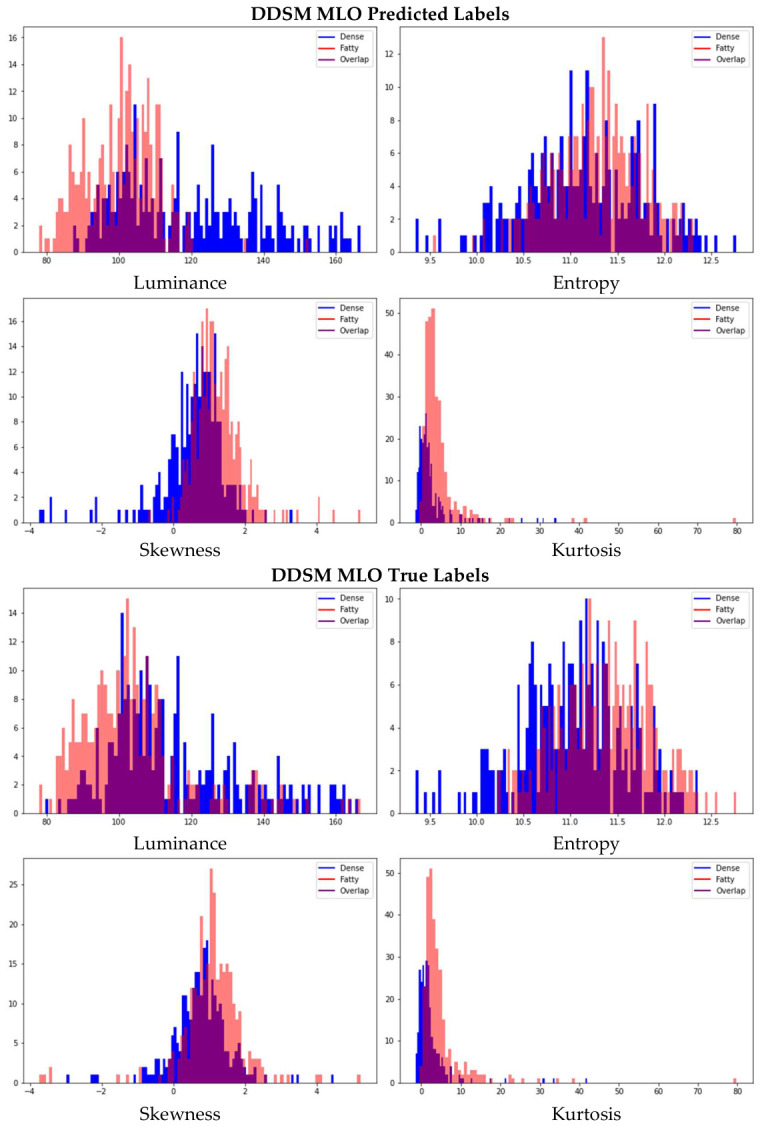
Label Quality Analysis Histograms for MLO Images of the DDSM Dataset.

**Figure A3:**
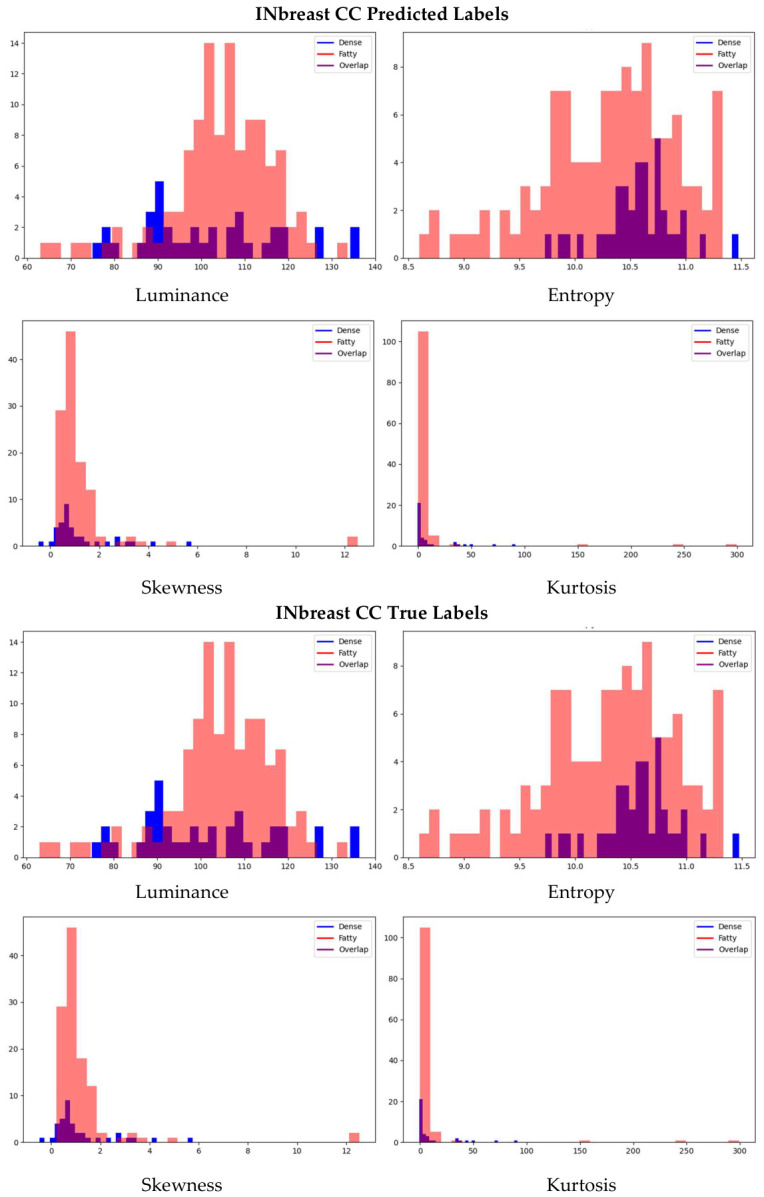
Label Quality Analysis Histograms for CC Images of the INbreast Dataset.

**Figure A4:**
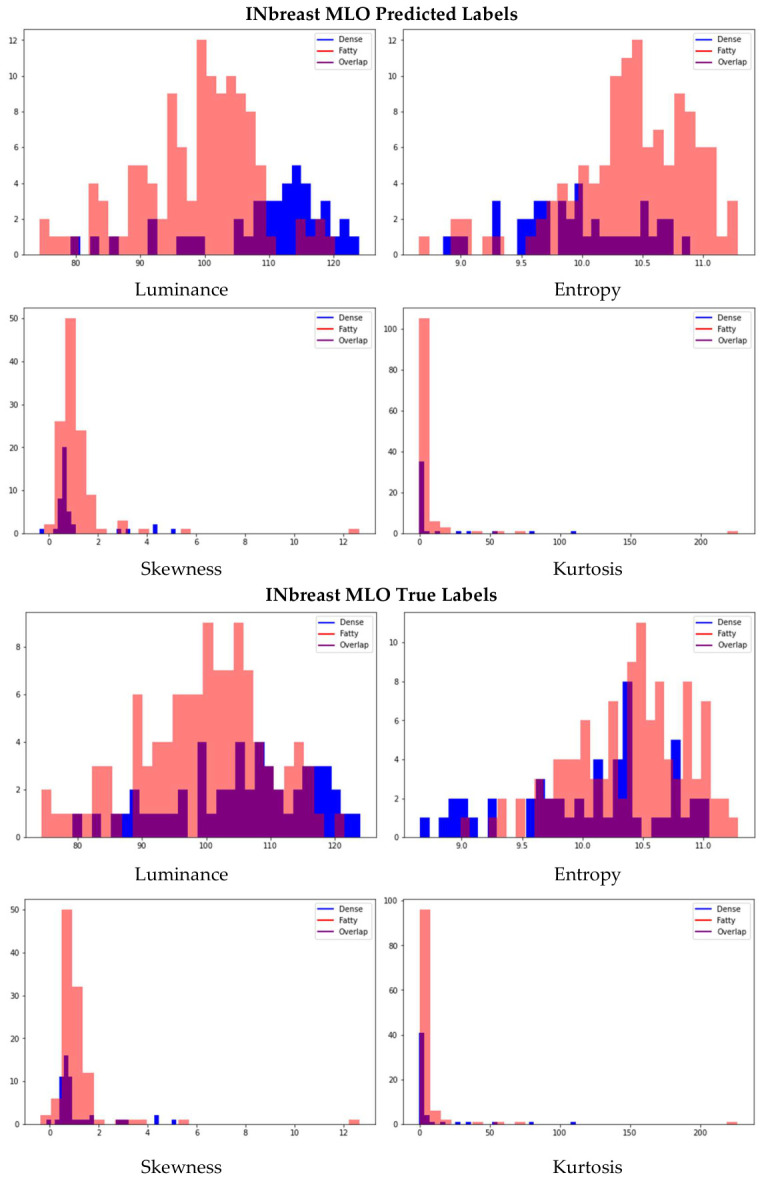
Label Quality Analysis Histograms for MLO Images of the INbreast Dataset.

This section presents the label quality analysis histograms for the CC and MLO images of the DDSM and INbreast datasets. It goes over the overlap or lack thereof between the two classes (Fatty and Dense), and explores the general trends exhibited by the framework. The histograms are shown in Figure A1, Figure A2, Figure A3 and Figure A4 in Appendix A.

In the DDSM dataset, the analysis demonstrates significant overlap between predicted and true labels for both CC and MLO images. Despite minor discrepancies, particularly in luminance and entropy features, the model’s predictions generally align with ground truth labels. Notably, the model tends to overestimate Fatty images and underestimate Dense ones, indicating a tendency towards false positives for Fatty classification. This is consistent with the unsupervised nature of the framework, which has no prior knowledge of class distributions.

Similarly, within the INbreast dataset, there is substantial agreement between the model’s predictions and expert-assigned labels, particularly for Fatty images. However, the model tends to predict more Fatty images and fewer Dense ones compared to expert assessment, consistent with the trends observed in DDSM. This behavior is most pronounced in the entropy and luminance features, where Dense distributions show a wider spread than their predicted counterparts.

Overall, while there are tendencies towards overprediction of Fatty images and underprediction of Dense ones across both datasets, the predicted and true distributions follow similar shapes across all four features. This suggests that the framework captures the underlying density structure of the data without supervision.

### 4.2. Pixel-Level Segmentation Evaluation

Segmentation quality was assessed for both DDSM and INbreast datasets using Silhouette Scores, WCSS, Davies–Bouldin Scores, and Calinski–Harabasz indices across CC and MLO views. The results are shown in Table 4.

Silhouette Scores were consistently high across all subsets, averaging between 0.9258 and 0.9376, indicating well-separated, cohesive clusters. Davies–Bouldin Scores were low (averages ranging from 0.0865 to 0.1011), further supporting strong clustering quality at the pixel level.

WCSS and Calinski–Harabasz indices showed wide value ranges, likely reflecting variability in image sizes. DDSM had higher average WCSS and Calinski–Harabasz values overall, but both datasets showed reasonable clustering quality, with averages significantly lower than their respective maximums. It should be noted that high pixel-level clustering metrics may partly reflect the intensity separability introduced by preprocessing rather than the strictly anatomical separation of fibroglandular and fatty tissue. Ground truth segmentation masks are not available for the datasets used in this study, precluding a pixel-level anatomical validation. However, the framework’s agreement with expert density labels across two independent datasets provides indirect evidence that the segmentation captures clinically meaningful density information.

### 4.3. Agreement with Ground Truth

This section presents the framework’s classification results following the discretization of the estimated density percentages and the correction process for the DDSM and INbreast datasets. The results are shown in Table 5.

Prior to the correction process, both the DDSM and INbreast datasets showed solid classification performance. For DDSM, CC images had an accuracy of 71.05% and a weighted F1-score of 70.58% (Fatty: 74.87%, Dense: 65.86%), while MLO images achieved 70.52% accuracy and a weighted F1-score of 70.83% (Fatty: 72.67%, Dense: 68.82%). Combined, DDSM results yielded an overall accuracy of 71.14% and a weighted F1-score of 71.05%, with AUC values of 0.71 across both continuous and discrete thresholds.

INbreast showed similar trends. Before correction, CC images had 71.14% accuracy and a weighted F1-score of 67.49% (Fatty: 81.22%, Dense: 37.68%), while MLO images reached 72.50% accuracy and a weighted F1-score of 72.93% (Fatty: 81.42%, Dense: 55.32%). Combined accuracy was 73.14%, with a weighted F1-score of 71.29%, and AUC values ranging from 0.60 to 0.68.

Following the correction process, only minor adjustments were needed: 34 corrections in DDSM CC and 6 in MLO. This led to a slight increase in CC accuracy to 71.46%, with improved F1 for the Dense class (68.05%) and a weighted F1 of 71.27%. MLO accuracy rose to 70.87%. Patient-level agreement in DDSM improved with the presence of more images, reaching 75.00% for patients with all four images, and the per-patient AUC remained at 0.71. In INbreast, just 2 corrections were made in each of the two subsets. The small number of corrections across both datasets is noteworthy: it suggests that the confidence metric precisely flags only genuinely ambiguous cases rather than broadly reassigning labels, while still yielding measurable accuracy gains. CC accuracy increased to 72.48%, with F1-scores rising to 82.10% (Fatty) and 40.58% (Dense), and a weighted F1 of 69.00%. MLO accuracy improved to 73.75%. Combined performance held at 73.14% accuracy and 71.29% weighted F1. Interestingly, per-patient agreement decreased with the presence of more images, from 79.28% (one image) to 72.72% (four images), though the overall per-patient AUC improved to 0.75. Balanced accuracy, computed as the average of per-class recall, was 71.38% for DDSM and 74.85% for INbreast at the per-patient level. This further suggests reasonably consistent performance across both classes despite the class imbalance.

Table 6 presents the classification accuracy broken down by original BI-RADS category for both datasets. Across both datasets, BI-RADS III consistently shows the lowest accuracy, with INbreast BI-RADS IV also performing notably below other categories.

**Table 6:** Agreement with the ground truth (%) per BI-RADS Category for DDSM and INbreast.

Dataset	Subset	BI-RADS I	BI-RADS II	BI-RADS III	BI-RADS IV
DDSM	CC	87.69	75.26	60.39	70.51
	MLO	77.14	73.21	60.36	79.00
	Combined (CC and MLO)	82.22	74.15	60.37	75.28
	Per-Patient	80.58	71.52	63.01	78.91
INbreast	CC	92.00	92.31	27.03	40.00
	MLO	96.30	74.07	52.50	41.67
	Combined (CC and MLO)	94.23	83.02	40.26	40.91
	Per-Patient	94.44	80.00	62.69	62.50

In summary, both datasets demonstrated strong agreement with expert labels, with minor corrections yielding modest performance gains. The models were particularly consistent for the Fatty class and maintained stable AUC values across conditions.

### 4.4. Assessment of Image-Level Clustering Quality

Clustering quality, i.e., the unsupervised clustering of images into fatty and dense categories, was evaluated using the Silhouette Coefficient, Davies–Bouldin Score, and Calinski–Harabasz Index. Metrics were computed separately for CC and MLO views using statistical image features, scaled via MinMax, including luminance, entropy, percentiles, skewness, and kurtosis. The results are presented in Table 7.

**Table 7:** Image-Level Clustering Quality Evaluation Results.

Dataset	View	Features		Silhouette	Davies–Bouldin	Calinski–Harabasz
		Number	Set			
DDSM	CC	1	Kurtosis	0.9484	0.0719	23,488.45
		2	Skewness, Kurtosis	0.8985	0.1434	10,437.01
		3	Skewness, Kurtosis, Entropy	0.8209	0.2563	4617.76
		9	All	0.5840	0.6807	832.41
	MLO	1	Kurtosis	0.9512	0.0704	27,744.17
		2	Skewness, Kurtosis	0.8947	0.1515	10,824.77
		3	Skewness, Kurtosis, Ranges	0.7976	0.2906	4170.39
		9	All	0.5505	0.7544	806.83
INbreast	CC	1	Kurtosis	0.9490	0.0566	1368.14
		2	Skewness, Kurtosis	0.9048	0.1035	672.78
		3	Skewness, Kurtosis, 25th Percentile	0.7858	0.2559	363.43
		9	All	0.4841	0.7703	79.26
	MLO	1	Kurtosis	0.9559	0.0876	3161.70
		2	Skewness, Kurtosis	0.9222	0.1349	1630.07
		3	Skewness, Kurtosis, 25th Percentile	0.8079	0.2833	802.84
		9	All	0.5157	0.8121	143.43

For DDSM, clustering performance was strong across both CC and MLO views. Using all features, CC images achieved a silhouette score of 0.5840, a Davies–Bouldin score of 0.6807, and a Calinski–Harabasz Index of 832.41, indicating solid cluster separation and cohesion. Feature-wise, kurtosis alone yielded the highest silhouette score (0.9484), while the combination of kurtosis and skewness also performed well (0.8985). Among three-feature combinations, skewness, kurtosis, and entropy were most effective (0.8209).

MLO images showed similar trends, with all features producing a silhouette score of 0.5505, a Davies–Bouldin score of 0.7544, and a Calinski–Harabasz Index of 806.83. Again, kurtosis alone gave the best silhouette score (0.9512), followed by kurtosis and skewness (0.8947), and a three-feature combination of skewness, kurtosis, and intensity ranges (0.7976).

For INbreast, CC images using all features produced a silhouette score of 0.4841, a Davies–Bouldin score of 0.7703, and a Calinski–Harabasz Index of 79.25. The highest silhouette score was again obtained using kurtosis alone (0.9490), followed by the kurtosis-skewness pair (0.9048). The most effective three-feature combination was skewness, kurtosis, and the 25th percentile (0.7858).

MLO images in INbreast showed slightly stronger clustering, with all features giving a silhouette score of 0.5157, a Davies–Bouldin score of 0.8121, and a Calinski–Harabasz Index of 143.43. Kurtosis alone again stood out (0.9559), while kurtosis and skewness yielded 0.9222. The best-performing trio, skewness, kurtosis, and the 25th percentile, achieved a silhouette score of 0.8079.

## 5. Discussion

### 5.1. Discussion of the Results

In this section, an analysis of the results presented in the previous section is provided for the two datasets, DDSM and INbreast. It summarizes the performance of the presented framework and sheds light on some of the difficulties it had in distinguishing between Fatty and Dense mammographic images. For both datasets, the framework demonstrates substantial agreement with expert-assigned labels and strong pixel-level segmentation quality. As noted in Section 4.2, pixel-level clustering metrics may partly reflect preprocessing-induced contrast rather than purely anatomical separation. Nonetheless, the consistent agreement with expert labels across both datasets suggests that the segmentation captures density-relevant structure, not just contrast enhancement. The tendency to overpredict Fatty images and underpredict Dense ones is consistent across all views and datasets, and is expected given that the framework has no prior knowledge of class distributions. A per-category breakdown of classification accuracy, presented in Table 6, confirms that BI-RADS III images are the primary source of misclassification across both datasets and views. For DDSM, BI-RADS III accuracy consistently falls in the 60–63% range, substantially below BI-RADS I (77–88%) and BI-RADS IV (70–79%). For INbreast, the pattern is more pronounced. BI-RADS III accuracy drops to 27.03% for CC images, and BI-RADS IV performance is similarly weak, ranging from 40 to 62% across subsets. This likely reflects the dataset’s severe class imbalance, where dense images constitute fewer than a third of the total. This stems from the inherent ambiguity of intermediate density cases, where the boundary between fatty and dense tissue is gradual rather than distinct. Notably, inter-observer variability among radiologists is also highest in this category, suggesting that the difficulty is not specific to unsupervised methods but is a fundamental challenge of the density estimation task itself. The framework’s strong performance on BI-RADS I and II cases, which represent the clearest fatty presentations, is consistent with its tendency to favor the Fatty class, as noted in Section 4.1. The decline in per-patient agreement with increasing numbers of images per patient in INbreast warrants further investigation. One possible explanation is that patients with more available views represent more complex or ambiguous cases, which are inherently harder to classify correctly without supervision.

Overall, the multifaceted evaluation confirms that the framework provides reliable unsupervised density estimates across two independent datasets. The consistent behavior across DDSM and INbreast, despite differences in dataset size and class distribution, suggests that the framework is not overtly dataset-specific, though broader generalizability would require validation on additional datasets. With the exception of the binarization threshold, adjusted per dataset to account for differences in acquisition and standardization, the preprocessing and segmentation pipeline is identical across DDSM and INbreast. Dataset-specific calibration is otherwise limited to α, β, CLAHE grid size, and the continuity loss function, all affecting only the binary classification stage. Several supervised state-of-the-art methods, as discussed in Section 5.2, do not demonstrate this cross-dataset consistency.

### 5.2. Comparison to the Literature

This section presents a comparison between the proposed framework and related studies. With the exception of one, all reviewed works use classification methods that differ significantly from ours. As such, agreement with ground truth is the only relevant basis for comparison. Given that our framework classifies mammograms into qualitative density classes using unsupervised thresholding rather than supervised learning, this comparison does not fully reflect its labeling quality. However, it remains useful for gauging how the framework performs relative to the current state-of-the-art. Table 8 presents a comparison of studies that conducted experiments using the DDSM and INbreast datasets.

With regard to studies utilizing DDSM, in [28], an accuracy of 96.35% was achieved using a linear kernel SVM on MLO images. Similarly, Ref. [40] reported a notable 84.17% accuracy on MLO images with a hierarchical hybrid model. Although both studies show stronger alignment with expert assessments for MLO images than our work, they each use smaller subsets—240 images in [28] and 480 in [40], compared to 563 in our experiments. Moreover, neither study provides metrics beyond accuracy, limiting the ability to fully evaluate model performance.

The work in [37] reports results for both CC and MLO images separately, with better performance on CC (85.10%) than MLO (66.95%). It should be noted that their selected subset is also significantly smaller, 200 images per view, whereas our work utilizes 487 CC and 563 MLO images. While their model performs well on CC images in terms of accuracy, F1-score, and AUC, our framework surpasses it by a clear margin on MLO images. Additionally, our model maintains consistent performance across metrics, in contrast to [37], where the F1-score notably lags behind what would be expected based on their accuracy and AUC results.

As for studies that conducted experiments on the INbreast dataset, the approach in [33] closely resembles our framework, employing deep-learning-based segmentation followed by arithmetic processing and thresholding to derive qualitative density labels. Their method clearly outperforms ours across all metrics. However, two key distinctions should be noted. First, the segmentation in [33] is supervised and trained on expert-labeled segmentations specific to the INbreast dataset, with experts directly involved in the study. This is significant for several reasons: manual annotation is both costly and labor-intensive; models trained exclusively on INbreast annotations may struggle to generalize; and, as previously discussed, expert labels are inherently subjective, with high inter-observer variability. In contrast, our framework avoids expert involvement entirely, making it both more cost-effective and less prone to subjective bias.

Second, Ref. [33] employs data augmentation to balance the dataset, increasing the number of images from 410 to 3192. While this likely enhances their model’s performance on INbreast, it also raises concerns about overfitting and reduced generalizability. The study in [36] similarly achieves a high accuracy of 98.00% through transfer learning, but also relies on augmentation, making it vulnerable to the same potential pitfalls.

The work in [37] presents separate results for CC, MLO, and the full dataset. Their model outperforms ours on the MLO subset and the combined dataset but underperforms on CC images. Unlike the stable performance of our framework, their results appear inconsistent, with F1-scores across subsets falling well below expected levels.

When compared to [39], our framework reports a lower AUC but higher accuracy and F1-score. This implies that while Ref. [39] may better distinguish between classes, our model achieves a stronger balance between precision and recall, resulting in fewer classification errors overall.

Overall, it is evident that state-of-the-art methods, most of which are supervised, show stronger alignment with ground truth labels across both datasets when compared to our framework. This outcome is expected, as these supervised models are trained directly on expert-provided annotations, whereas our segmentation and density estimation pipeline operates without label supervision. Still, even the top-performing models exhibit notable limitations. Many of them either evaluate performance on smaller, less challenging subsets of data or utilize augmentation techniques, thereby generating large numbers of highly similar images with identical labels. While such practices may boost accuracy on a given dataset, they come at the cost of reduced generalizability.

It is also worth emphasizing that the highest-performing model, detailed in [33], depends on hand-annotated tissue segmentations, a resource-intensive and time-consuming solution. In contrast, our framework avoids the need for expert input at any stage of its core pipeline and maintains consistent performance across two independent datasets, suggesting practical potential as an annotation-free density estimation tool.

### 5.3. Ablation Study

An ablation study was conducted in order to evaluate the impact of individual components within the framework. Specifically, the impact of CLAHE, the utility of the correction process, and the need for adaptively sized ROIs were assessed. Additionally, the segmentation model was compared to Otsu thresholding, triangle thresholding, and K-means clustering.

#### 5.3.1. Impact of CLAHE

**Figure 9:**
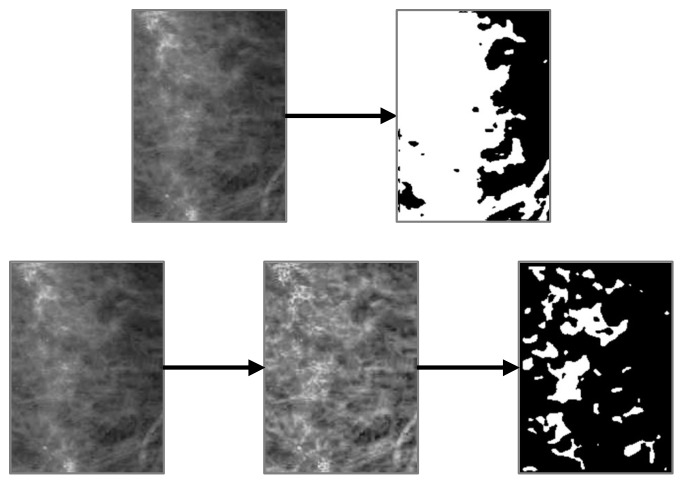
Sample segmentation results with and without applying CLAHE.

The application of CLAHE prior to segmentation has a measurable impact on framework performance. Figure 9 shows segmentation results of the same image with and without CLAHE applied to the original image. The difference between the two segmentation outputs is substantial, with direct implications for the quality of the resulting density estimates.

The framework implements CLAHE as a crucial step prior to segmentation. To measure the impact of CLAHE on the overall results, the framework was tested with the CLAHE step removed, and the results are summarized in Table 9. The results for DDSM are significantly better when CLAHE is applied. For INbreast, the impact of omitting CLAHE is comparatively minor, which may reflect inherent differences in image contrast between the two datasets. INbreast images may already exhibit more consistent contrast, whereas DDSM images appear more variable in this regard. In any case, the addition of CLAHE results in better framework performance on the two datasets, which makes it an indispensable part of the framework.

CLAHE parameters, such as the contrast limiting threshold, can be changed based on the requirements of an application. The contrast limiting threshold was set to 2.0 empirically, but the histogram equalization (HE) grid size required more testing. For the DDSM dataset, better performance was attained with a grid size of 8 × 8, but the performance was comparable with a grid size of 16 × 16. In fact, the performance on MLO images in particular was better with a grid size of 8 × 8. For INbreast, by contrast, the performance was notably better with a grid size of 16 × 16. A summary of the results for the two grid sizes is provided in Table 10.

#### 5.3.2. Effects of Correction

The correction process is implemented following the discretization of computed percentage densities into two classes (Fatty and Dense). This is only done for images where the confidence metric is set to “Not Confident,” in order to account for any potential segmentation errors that may result from the almost entirely homogeneous appearance of some images. Applying it has a noticeable impact on classification performance. The results with and without correction are displayed in Table 11. As can be seen, while the gains following correction are modest, the improvements in accuracy and F1-score across individual subsets are consistent and meaningful. Notably, INbreast per-patient accuracy is unchanged, suggesting that correction primarily benefits image-level classification rather than patient-level aggregation.

#### 5.3.3. Standard vs. Adaptive ROIs

Initially, ROIs were extracted from the central regions of breast mammograms with a fixed size of 128 × 128 pixels. Adaptive ROI sizing was subsequently introduced to address the limitations of this approach. Testing was performed to quantify the difference in performance with and without adaptive ROI sizing. The results are presented in Table 12. The results indicate that the overall performance is considerably better when the adaptive ROIs are utilized. Per-patient assessment quality improves substantially in accuracy and F1-score for both datasets.

#### 5.3.4. Other Segmentation Models

The segmentation algorithm from [15] demonstrates strong performance across both datasets. However, it is possible to replace it with other unsupervised segmentation methods, whether to streamline the framework, create a baseline to compare performance, or evaluate alternative approaches. Three segmentation methods were introduced as potential replacements for the algorithm in [15]: Otsu thresholding, triangle thresholding, and K-means clustering. The results from experiments using each of the segmentation alternatives on the two datasets are presented and contrasted with the algorithm from [15] in Table 13 and Table 14.

For DDSM, the classification accuracy and F1-score resulting when using the algorithm from [15] are highest by a notable margin. In terms of unsupervised labeling quality, the algorithm also results in the best silhouette, Davies–Bouldin, and Calinski–Harabasz scores for CC images, but is minimally outperformed by triangle thresholding in both Davies–Bouldin and Calinski–Harabasz scores for MLO images. Given that difference in overall accuracy is quite large (over 5% in the per-patient scores), the difference in labeling quality in MLO images is negligible.

For INbreast, the per-patient accuracy produced by the algorithm from [15] is matched by K-means clustering, and the F1-score is slightly exceeded. It is also outperformed in terms of labeling quality on MLO images. That a simple clustering approach matches the performance of a deep learning segmentation algorithm on INbreast warrants consideration. Given its considerably weaker performance on DDSM, this may reflect dataset-level differences in image complexity or inter-class separability rather than a genuine equivalence between the two methods. In any case, the overall accuracy of the K-means model drops to 77.88% when at least 2 images are available per patient, and further down to 71.11% when all 4 images are available, compared to 78.43% and 72.73%, respectively, using the algorithm from [15]. As such, it can be concluded that, even at the cost of additional framework complexity, it is still worth using the deep-learning segmentation algorithm.

### 5.4. Limitations and Future Work

Several limitations of the presented framework should be acknowledged. The framework has only been tested on two publicly available datasets. Its applicability to mammograms from different acquisition systems, image resolutions, or patient populations has not been tested. Additionally, the selection of hyperparameters was conducted on a subset of 50 images per dataset. Those images were drawn from the same data used for the final evaluation. A formally held-out validation set would provide strong guarantees against optimistic bias. The framework also exhibits a consistent tendency to favor the Fatty class. This is an inherent consequence of operating without prior knowledge of class distribution, and is most notable for BI-RADS III cases. The binary classification scheme further compounds this issue by placing the most ambiguous, intermediate-density class at the class boundary. Finally, the absence of ground truth segmentation masks in both datasets precludes an anatomical validation at the pixel level. Further, the continuous density estimates have not yet been validated against volumetric measurements or direct radiologist assessment.

For future work, alternative unsupervised segmentation methods should be explored to determine whether stronger segmentation backbones can improve density estimates. The confidence metric and label correction mechanism could be further refined, and the ROI extraction approach could be evaluated against other region selection strategies. Statistical significance testing, such as McNemar’s test, would help determine whether performance improvements from the correction process and adaptive ROI sizing are statistically meaningful. A collaboration with medical professionals would also help enable clinical validation of the framework’s density estimates against radiologist assessments and volumetric measurements. Finally, the framework should be evaluated using a formally held-out validation set to provide stronger guarantees against optimistic bias in the reported results.

## 6. Conclusions

In this work, a framework for breast density estimation based on unsupervised segmentation of mammographic images was proposed. The framework introduces preprocessing techniques for breast reorientation, artefact removal, and adaptive ROI extraction, along with a CNN-based segmentation pipeline tuned for mammographic density separation. A binary confidence metric was proposed to identify unreliable segmentation outputs, with a correction mechanism applied to low-confidence cases. A confidence-filtered majority voting scheme was introduced for per-patient density classification.

The framework was evaluated on two public datasets, DDSM and INbreast, across four phases: label quality analysis, pixel-level segmentation evaluation, agreement with expert labels, and image-level unsupervised clustering quality assessment. Segmentation quality was consistently strong, with Silhouette scores exceeding 0.92 across all subsets. Agreement with expert labels reached 71.43% and 79.28% for DDSM and INbreast, respectively, achieved using a core pipeline that requires no supervision. The clustering quality assessment confirmed effective unsupervised labeling, and further testing demonstrated that each module in the framework contributes positively to overall classification performance. In summary, the proposed framework is a practical and non-subjective approach for quantitative breast density estimation that requires no expert annotation in its core pipeline.

## Figures and Tables

**Table 1:** Summary of image and patient counts per dataset and view following selection and preprocessing.

Dataset	View	Images	Fatty	Dense	Patients
DDSM	CC	487	255	232	805
	MLO	563	294	269	
	Total	1050	549	501	
INbreast	CC	149	102	47	111
	MLO	160	108	52	
	Total	309	210	99	

**Table 2:** Summary of Results of the Proposed Framework.

Dataset	Pixel-Level Segmentation Quality			Image-Level Unsupervised Clustering Quality			Agreement with Ground Truth (Per-Patient)	
	Metric	Scores		Metric	Scores		Metric	Scores (%)
		CC	MLO		CC	MLO		
DDSM	SC	0.926	0.936	SC	0.584	0.551	Acc	71.43
	WCSS	16.8 M	21.5 M					
	DB	0.111	0.09	DB	0.681	0.754	F1	71.4
	CH	36.6 K	54.4 K	CH	832.4	806.8		
INbreast	SC	0.935	0.938	SC	0.484	0.516	Acc	79.28
	WCSS	10.3 M	8.2 M					
	DB	0.09	0.09	DB	0.770	0.812	F1	79.02
	CH	27 K	25.1 K	CH	79.25	143.4		

**Table 3:** Optimized values for α and β.

Dataset	View	α	β
DDSM	CC	−0.32	−1.10
	MLO	−0.47	−1.90
INbreast	CC	0.77	−0.60
	MLO	0.75	−1.60

**Table 4:** Pixel-Level Segmentation Evaluation Results.

Dataset		Metric	Average	Minimum	Maximum
DDSM	CC	SC	0.9258	0.8371	0.9815
		WCSS	16,854,724.51	197,430.51	144,346,541.33
		DB	0.1011	0.0285	0.2342
		CH	36,581.62	0.0066	496,561.51
	MLO	SC	0.9355	0.8485	0.9875
		WCSS	21,591,404.50	1,252,671.60	185,403,996.53
		DB	0.0923	0.0195	0.2674
		CH	54,360.20	0.2794	978,069.43
INbreast	CC	SC	0.9350	0.8755	0.9898
		WCSS	10,291,134.81	481,094.11	41,829,238.01
		DB	0.0904	0.0166	0.2033
		CH	26,952.22	0.5716	160,076.65
	MLO	SC	0.9376	0.8675	0.9882
		WCSS	8,183,997.50	618,902.53	37,745,631.35
		DB	0.0865	0.0167	0.1935
		CH	25,081.47	64.55	81,071.36

**Table 5:** Classification results demonstrating agreement with ground truth (BI-RADS classes).

Dataset	Subset		Precision	Recall	F1-Score (%)	Accuracy (%)	AUC
DDSM	CC	Fatty	0.7042	0.7843	74.21	71.46	0.7111
		Dense	0.7279	0.6379	68.05		
		Overall	0.7161	0.7146	71.27		
	MLO	Fatty	0.7124	0.7415	72.67	70.87	0.7072
		Dense	0.7043	0.6729	68.82		
		Overall	0.7085	0.7087	70.83		
	Combined (CC and MLO)	Fatty	0.7085	0.7614	73.40	71.14	0.7090
		Dense	0.7152	0.6567	68.47		
		Overall	0.7117	0.7114	71.05		
	**Per-Patient**	Fatty	0.7136	0.7379	72.55	**71.43**	**0.7137**
		Dense	0.715	0.6896	70.21		
		Overall	0.7143	0.7143	71.40		
INbreast	CC	Fatty	0.7402	0.9216	82.10	72.48	0.6097
		Dense	0.6364	0.2979	40.58		
		Overall	0.7074	0.7248	69.00		
	MLO	Fatty	0.7797	0.8519	81.42	73.75	0.6759
		Dense	0.6190	0.5000	55.32		
		Overall	0.7275	0.7375	72.93		
	Combined (CC and MLO)	Fatty	0.7592	0.8857	81.76	73.14	0.6449
		Dense	0.6250	0.4040	49.08		
		Overall	0.7162	0.7314	71.29		
	**Per-Patient**	Fatty	0.8354	0.8684	85.16	**79.28**	**0.7485**
		Dense	0.6875	0.6286	65.67		
		Overall	0.7888	0.7903	79.02		

**Table 8:** Comparison of the proposed framework with related works on the DDSM and INbreast datasets. Entries marked with “-” indicate metrics not reported in the original study. For our framework, Precision, Recall, F1-Score, and Accuracy are weighted averages across Fatty and Dense classes.

Study	Subset	Model	Prec. (%)	Rec. (%)	F1 (%)	Acc. (%)	AUC
DDSM							
X. Gong et al., 2019 [28],	MLO, 240 Images	SVM (Linear Kernel)	-	-	-	96.35	-
I. Kumar et al., 2017 [40],	MLO, 480 Images	Hybrid—SVM/NFC/KNN	-	-	-	84.17	-
W. Zhao et al., 2021 [37],	CC, 200 Images	BASCNet	-	-	73.92	85.10	0.92
	MLO, 200 Images		-	-	49.84	66.95	0.83
Ours	CC, 487 Images	CNN + Arithmetic Division	71.61	71.46	71.27	71.46	0.7111
	MLO, 563 Images		70.85	70.87	70.83	70.87	0.7072
	Combined, 1050 Images		71.17	71.14	71.05	71.14	0.7090
	Per-Patient, 805 Patients		71.43	71.43	71.40	71.43	0.7137
INbreast							
N. Saffari et al., 2020 [33],	Combined, 3192 Images	cGAN-UNet	97.85	97.85	-	98.62	-
B. Mohammed and B. Najida, 2021 [36],	Combined, 1868 Images	Inception_ ResNet_V2	97.00	96.00	96.00	98.00	-
W. Zhao et al., 2021 [37],	CC	BASCNet	-	-	46.93	70.41	0.86
	MLO		-	-	68.09	86.27	0.94
	Combined, 410 Images		-	-	78.11	90.51	0.99
C. Li et al., 2021 [39],	Combined 410 Images	ResNet50	-	-	63.50	70.00	0.84
Ours	CC, 149 Images	CNN + Arithmetic Division	70.74	72.48	69.00	72.48	0.61
	MLO, 160 Images		72.75	73.75	72.93	73.75	0.68
	Combined, 309 Images		71.62	73.14	71.29	73.14	0.65
	Per-Patient, 111 Patients		78.88	79.03	79.02	79.28	0.75

**Table 9:** Comparison of results with and without applying CLAHE.

CLAHE	Dataset		Accuracy (%)	F1-Score (%)
Included	DDSM	CC	71.46	71.27
		MLO	70.87	70.83
		Per-Patient	71.43	71.40
	INbreast	CC	72.48	69.00
		MLO	73.75	72.93
		Per-Patient	79.28	79.02
Excluded	DDSM	CC	60.58	60.11
		MLO	54.92	54.94
		Per-Patient	58.66	58.76
	INbreast	CC	69.33	66.67
		MLO	70.63	70.55
		Per-Patient	78.38	78.38

**Table 10:** Comparison of results using different CLAHE grid sizes.

CLAHE Grid Size	Dataset		Accuracy (%)	F1-Score (%)
8 × 8	DDSM	CC	71.46	71.27
		MLO	70.87	70.83
		Per-Patient	71.43	71.40
	INbreast	CC	67.57	66.40
		MLO	63.46	64.09
		Per-Patient	63.06	64.13
16 × 16	DDSM	CC	68.33	67.94
		MLO	72.08	72.06
		Per-Patient	71.11	71.09
	INbreast	CC	72.48	69.00
		MLO	73.75	72.93
		Per-Patient	79.28	79.02

**Table 11:** Comparison of results with and without the correction process.

Correction	Dataset		Accuracy (%)	F1-Score (%)
Omitted	DDSM	CC	71.05	70.58
		MLO	70.52	70.45
		Per-Patient	70.81	70.76
	INbreast	CC	71.14	67.49
		MLO	72.50	71.41
		Per-Patient	79.28	79.02
Performed	DDSM	CC 34/487 Corrected Instances	71.46	71.27
		MLO 4/563 Corrected Instances	70.87	70.83
		Per-Patient	71.43	71.40
	INbreast	CC 2/149 Corrected Instances	72.48	69.00
		MLO 2/160 Corrected Instances	73.75	72.93
		Per-Patient	79.28	79.02

**Table 12:** Comparison of results using standard and adaptive ROI sizes.

ROI Size	Dataset		Accuracy (%)	F1-Score (%)
Standard (128 × 128)	DDSM	CC	67.62	67.59
		MLO	66.55	66.19
		Per-Patient	68.53	68.43
	INbreast	CC	71.14	65.15
		MLO	70.15	69.95
		Per-Patient	72.42	68.09
Adaptive	DDSM	CC	71.46	71.27
		MLO	70.87	70.83
		Per-Patient	71.43	71.40
	INbreast	CC	72.48	69.00
		MLO	73.75	72.93
		Per-Patient	79.28	79.02

**Table 13:** Comparison of classification results using different segmentation methods.

Segmentation Method	Dataset		Accuracy (%)	F1-Score (%)
Otsu Thresholding	DDSM	CC	62.88	62.63
		MLO	58.80	58.20
		Per-Patient	61.41	61.31
	INbreast	CC	70.20	69.73
		MLO	72.67	71.85
		Per-Patient	75.68	75.77
Triangle Thresholding	DDSM	CC	65.72	65.71
		MLO	67.78	67.76
		Per-Patient	66.22	66.16
	INbreast	CC	72.19	70.70
		MLO	70.81	69.60
		Per-Patient	74.77	74.77
K-means	DDSM	CC	63.69	62.78
		MLO	58.98	58.50
		Per-Patient	61.90	61.68
	INbreast	CC	72.85	69.54
		MLO	72.05	71.83
		Per-Patient	79.28	79.20
Algorithm from [15]	DDSM	CC	71.46	71.27
		MLO	70.87	70.83
		Per-Patient	71.43	71.40
	INbreast	CC	72.48	69.00
		MLO	73.75	72.93
		Per-Patient	79.28	79.02

**Table 14:** Comparison of image-level clustering quality using different segmentation methods.

Segmentation Method	Dataset		Silhouette	Davies–Bouldin	Calinski–Harabasz
Otsu Thresholding	DDSM	CC	0.5081	0.8166	583.91
		MLO	0.5155	0.8340	674.53
	INbreast	CC	0.4606	0.9150	115.18
		MLO	0.5561	0.6803	211.83
Triangle Thresholding	DDSM	CC	0.5159	0.7913	644.51
		MLO	0.5459	0.7444	839.94
	INbreast	CC	0.4786	0.8273	120.07
		MLO	0.5110	0.8011	155.00
K-means	DDSM	CC	0.5051	0.8208	538.22
		MLO	0.5158	0.8304	685.12
	INbreast	CC	0.4680	0.8860	77.27
		MLO	0.5394	0.7280	206.62
Algorithm from [15]	DDSM	CC	0.5840	0.6807	832.41
		MLO	0.5505	0.7544	806.83
	INbreast	CC	0.4841	0.7703	79.25
		MLO	0.5157	0.8121	143.43

## Data Availability

The data presented in this study are available in the Cancer Imaging Archive at https://www.cancerimagingarchive.net/collection/cbis-ddsm/, reference number [16], and at https://www.kaggle.com/datasets/ramanathansp20/inbreast-dataset, reference number [17]. These data were derived from the following resources available in the public domain: the Curated Breast Imaging Subset of DDSM (CBIS-DDSM), available at https://www.cancerimagingarchive.net/collection/cbis-ddsm/ (accessed on 45 June 2026), and the INbreast dataset, available at https://www.kaggle.com/datasets/ramanathansp20/inbreast-dataset (accessed on 45 June 2026).
